# Reinforced liquid state machines—new training strategies for spiking neural networks based on reinforcements

**DOI:** 10.3389/fncom.2025.1569374

**Published:** 2025-05-23

**Authors:** Dominik Krenzer, Martin Bogdan

**Affiliations:** ^1^Neuromorphic Information Processing, Leipzig University, Leipzig, Germany; ^2^Center for Scalable Data Analytics and Artificial Intelligence, Leipzig, Germany; ^3^School of Embedded Composite Artificial Intelligence, Leipzig, Germany

**Keywords:** spiking neural networks, bio-inspired learning, reinforced-spike-timing-dependent plasticity (R-STDP), speech recognition, adaptive neural architectures, neuromorphic computing, temporal learning

## Abstract

**Introduction:**

Feedback and reinforcement signals in the brain act as natures sophisticated teaching tools, guiding neural circuits to self-organization, adaptation, and the encoding of complex patterns. This study investigates the impact of two feedback mechanisms within a deep liquid state machine architecture designed for spiking neural networks.

**Methods:**

The Reinforced Liquid State Machine architecture integrates liquid layers, a winner-takes-all mechanism, a linear readout layer, and a novel reward-based reinforcement system to enhance learning efficacy. While traditional Liquid State Machines often employ unsupervised approaches, we introduce strict feedback to improve network performance by not only reinforcing correct predictions but also penalizing wrong ones.

**Results:**

Strict feedback is compared to another strategy known as forgiving feedback, excluding punishment, using evaluations on the Spiking Heidelberg data. Experimental results demonstrate that both feedback mechanisms significantly outperform the baseline unsupervised approach, achieving superior accuracy and adaptability in response to dynamic input patterns.

**Discussion:**

This comparative analysis highlights the potential of feedback integration in deepened Liquid State Machines, offering insights into optimizing spiking neural networks through reinforcement-driven architectures.

## 1 Introduction

Neuromorphic computing (Schuman et al., 2022) aims to replicate the principles of information processing found in the brain, a system known for its remarkable efficiency and adaptability. The human brain can deal with vast amounts of sensory data, perform complex tasks like pattern recognition, decision making, and motor control, all while continuously learning and adapting. This efficiency is driven by the brains architecture, where billions of neurons communicate via discrete electrical impulses or spikes (Gerstner, 2001). These spikes are sparse in time, event-driven, and highly energy-efficient, making the brain's approach to computation vastly different from traditional digital processors or even Artificial Neural Networks (ANNs).

Inspired by the brains architecture, Spiking Neural Networks (SNNs) have emerged as a promising approach for neuromorphic information processing (Gerstner and Kistler, 2002). In contrast to conventional ANNs, which rely mainly on continuous activations, SNNs operate using binary, spike-based communication between neurons. This enables more biologically realistic computations (Maass, 1997), where neurons remain mostly inactive and only fire in response to significant changes in their input. Such event-driven behavior can conserve energy especially in combination with neuromorphic hardware implementations (Young et al., 2019). SNNs, in theory, can replicate the temporal dynamics and adaptive capabilities of the brain, making them an ideal candidate for low-power, high-performance architectures designed to handle complex, real-world data (Yamazaki et al., 2022).

One of the main challenges limiting the widespread adoption of SNNs is their difficulty in training (Tavanaei et al., 2019). In traditional ANNs, gradient-based methods like backpropagation have proven extreme effectiveness, allowing models to optimize complex objective functions across multiple layers. However, SNNs present unique challenges for gradient-based training. The discrete, non-differentiable nature of the spike events makes it difficult to directly apply backpropagation in the same way as ANNs (Neftci et al., 2019).

This has provoked research into how feedback might operate in SNNs and in the brain more broadly (Ororbia, 2023). One promising approach to training SNNs is e-prop (Bellec et al., 2020), a biologically plausible alternative to traditional backpropagation through time (BPTT). This framework leverages biologically realistic mechanisms, like eligibility traces and feedback signals to train recurrent SNNs. Recurrent SNNs are highly promising due to their ability to retain memory and context, making them uniquely suited for tasks involving sequential data, temporal dependencies, and contextual understanding. This capability mirrors biological neural circuits, where recurrency is fundamental for processing. However, training reccurent SNNs remains challenging due to to vanishing or exploding gradients, which hinder the learning of long-range dependencies.

To better understand and leverage recurrent SNNs, it is essential to separate the effects of recurrency in maintaining memory and temporal dynamics from the learning signals that guide weight updates (Evanusa et al., 2024). Inherent recurrency enables networks to process and store information over time, forming the basis of their memory capabilities. On the other hand, learning signals, which drive synaptic changes, should be treated as distinct mechanisms that influence how memory is updated or retained based on task-relevant feedback. Decoupling these functions can simplify network design and allow for more effective and scalable training methods that preserve the temporal processing power of recurrent SNNs.

To address the issue of how feedback operates in that kind of setting, we use a novel approach that introduces a non-backpropagation-based method for incorporating feedback in SNNs, called the Reinforced Liquid State Machine (RLSM) (Krenzer and Bogdan, 2025). The method leverages the idea of predictive coding (Rao and Ballard, 1999) to incorporate feedback in a multilayer Liquid State Machine (LSM) architecture (Maass, 2011). The RLSM is a computational framework where the system generates predictions about incoming sensory input and continually compares these predictions with actual sensory data. When predictions do not match the input, prediction errors are generated and sent back up the hierarchy to adjust and refine the predictions. This iterative process of minimizing prediction errors allows the architecture to update its internal models and improve sensory interpretation over time.

The primary focus of this study is to compare two types of feedback mechanisms in the RLSM. The first utilizes feedback solely based on a positive reward signal to modulate synaptic plasticity, reinforcing correct predictions like in Krenzer and Bogdan (2025). The second, new introduced strict feedback, combines both reward and punishment, where synapses are strengthened for correct predictions and weakened for incorrect ones, providing a more refined mechanism for synaptic adjustment. A statistical analysis is conducted to evaluate the performance of these feedback systems in a series of experiments using the Spiking Heidelberg Digits dataset (SHD) (Cramer et al., 2022) for spoken digit recognition, highlighting the effects of each approach on learning and accuracy.

## 2 Related work

### 2.1 Liquid state machines

The LSM is a computational model that represents a class of SNNs designed to process data in a neurological plausible way. Introduced by Maass et al. (2002), LSMs are particularly noted for their ability to handle real-time processing tasks, such as speech recognition (Deckers et al., 2022), dynamic pattern recognition (Woo et al., 2024), and robotic control (de Azambuja et al., 2017). They operate on the principle of transforming a time-varying input signal into a high-dimensional, dynamic state space, often referred to as the liquid state. This state is generated by a recurrent neural network composed of spiking neurons. The behavior of the liquid is characterized by its sensitivity to input variations and its ability to retain a fading memory of past inputs. In essence, the liquid functions as a complex filter (El-Laithy and Bogdan, 2012), mapping the input stream into a rich, nonlinear representation. Unlike traditional feedforward neural networks, LSMs do not require extensive training of the reservoir component, as the liquids intrinsic recurent dynamics are naturally suited to capture the temporal correlations in the input data. Instead, only the readout layer requires training, which can be efficiently achieved using relatively simple learning algorithms, such as linear regression in our case. This separation of computation into a dynamic reservoir and a trainable readout layer is a defining characteristic of LSMs and underpins their versatility and efficiency. However, a single reservoir in reservoir computing can struggle to capture the layered dependencies present in hierarchical data structures (Ma et al., 2017). In speech data like in the Heidelberg Dataset (Cramer et al., 2022), for example, sounds combine to form phonemes, which in turn form words, phrases, and sentences, each with its own temporal dependencies. This hierarchical organization means that critical patterns exist at multiple levels, from micro-level sound features to macro-level semantic structures. Using multiple reservoirs allows the model to separately handle these layers, capturing both fine-grained acoustic features and broader linguistic patterns, which improves the accuracy and robustness of speech recognition tasks (Ma et al., 2017).

### 2.2 Deep liquid state machines

The study of Soures and Kudithipudi (2019) introduces the Deep Liquid State Machines (D-LSM), which uses multiple liquid layers inspired by deep learning. The D-LSM mimics the hierarchical processing observed in cortical structures, where layers of neurons engage in progressively complex feature extraction. The architecture consists of multiple liquid layers that are designed to capture and transform input signals into progressively more abstract representations. Between these layers a winner-takes-all network (WTA) (Oster et al., 2009) is encoding features by transforming the high dimensional code of liquids into a low dimensional representation. The WTA is driven by unsupervised competitive learning, meaning that each neuron is trying to represent certain spatial-temporal features by adapting its synaptic connection with a local hebbian learning rule. In this configuration, the WTA and encoding layers act as consecutive nonlinear filters, potentially leading to challenges where errors in local learning processes accumulate over time. To address this, we aim to incorporate neuromodulatory mechanisms into our work. For example, dopamine neurons, as described by Schultz (1998), generate predictive reward signals that help the brain correct errors by reinforcing accurate predictions and suppressing inaccurate ones. By incorporating such mechanisms, the idea is to enhance error correction and adaptability in our proposed learning architecture.

### 2.3 Neuromodulated spike-timing-dependent plasticity

Neuromodulated (Reinforced) Spike-Timing-Dependent Plasticity (R-STDP) (Frémaux and Gerstner, 2016) extends the classic concept of Spike-Timing-Dependent Plasticity (STDP) (Markram et al., 2012), a biological mechanism that describes adjustment of synaptic strength based on the precise timing of spikes between pre- and postsynaptic neurons. While STDP is governed by the relative timing of these spikes, leading to either long-term potentiation (LTP) or long-term depression (LTD) (Timothy V.P. Bliss, 2011) of the synapse, R-STDP incorporates the influence of neuromodulators—chemical signals like dopamine, serotonin, or acetylcholine into the plasticity process. In R-STDP, the presence of neuromodulators acts as a third factor that modulates the strength and direction of synaptic changes. This additional layer of modulation allows for more context-sensitive and task-specific learning (Juarez-Lora et al., 2022), making the synaptic updates not only depending on the timing of spikes but also on the global or local neuromodulatory signals. These signals reflect the overall state of the system or its environment. This three-factor framework bridges the gap between synaptic plasticity and higher-level learning processes, providing a biologically plausible mechanism for how experiences and environmental factors can dynamically influence learning. In the RLSM, a neuromodulator signal is introduced to mitigating error accumulation in the winner-takes-all layer and therefore improve learning capabilities.

In summary, while the LSM effectively leverages neurological principles, it lacks an inherent capacity for hierarchical data representation. A possible enhancement is the D-LSM, which introduces a way of adding deepness to the LSM paradigm. However, the information aggregation between this hierarchical reservoirs is only based local information. Therefore, they may lack the necessary global guidance to ensure that their adjustments move in the right direction. To address this, we introduce a novel reward system with R-STDP synapses that not only reinforces learning when the networks predictions are correct but also penalizes connections when they are incorrect.

## 3 Method

**Figure 1:**
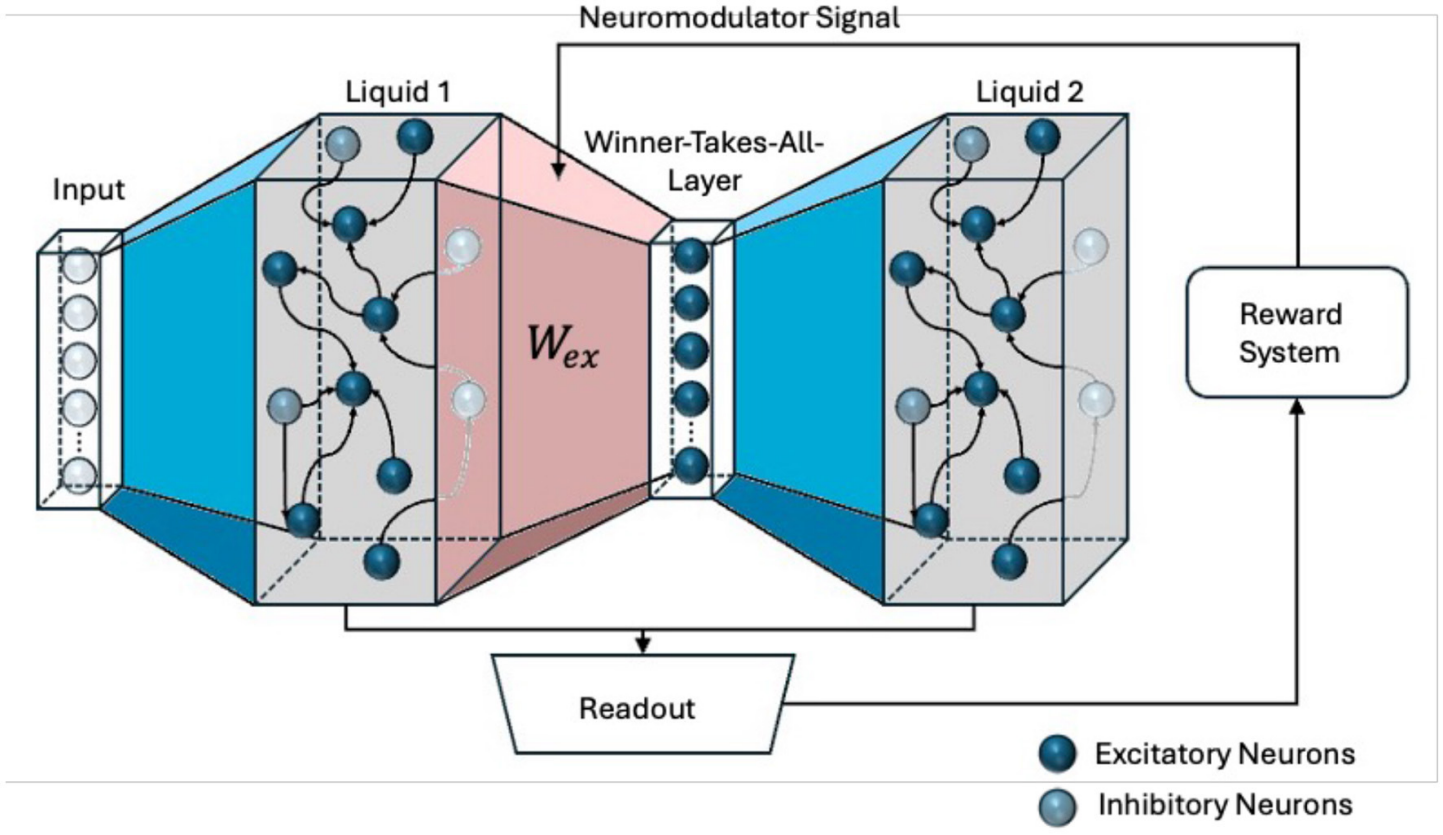
The Reinforced Liquid State Machine (RLSM) architecture. Two liquid layers are connected via a winner-takes-all layer. Trainable red weights *W*_ex_ link the first liquid layer to the winner layer. The readout output is compared to the true digit, feeding back into the weight update mechanism for *W*_ex_, driving adaptive learning in the system.

To investigate different feedback mechanisms, the RLSM learning architecture, shown in Figure 1, is utilized. It features two liquid layers, each functioning as a distinct reservoir of spiking neurons with dynamic synapses. These layers independently transform incoming temporal input streams into high-dimensional, nonlinear representations. Each liquid layer is supposed to capture different aspects or features of the input data, creating a diverse set of dynamic states. Between these liquid layers is an interposed WTA, which plays a crucial role in filtering and selecting the most salient features from the outputs of the liquid layers. A WTA implements a competitive mechanism where only the most strongly activated neurons are allowed to propagate their signals at a particular time to the next stage of the network. Those neurons are representing the most relevant features or patterns at that moment.

The architecture aligns with the framework of predictive coding (Rao and Ballard, 1999), wherein the WTA generates sensory predictions based on the input it receives from the preceding liquid layer. These predictions are encoded in the spiking activity of the WTA neurons, which represent the most likely interpretation of the incoming signals. Feedback based on the performance of the whole system serves as a top-down modulation signal, correcting discrepancies between the WTAs predictions and the actual input dynamics. This top-down feedback, coupled by a reward signal, modulates the synaptic weights of the WTA layer through R-STDP, adjusting its predictions to better align with the temporal patterns of the input data. This iterative process, where predictions are generated by the WTA layer and updated by top-down signals, reflects the core principle of predictive coding: minimizing the prediction error through hierarchical feedback loops.

### 3.1 Liquid configuration

Similar to the setup in Maass et al. (2002) the liquid network comprises of 135 recurrent connected neurons and dynamic synapses. The neuronal dynamics are described by a basic Leaky Integrate and Fire neuron (LIF) modeling the membrane potential *V* depending on time *t*:


(1) $$\begin{array}{l} \tau_{m}\frac{dV\left(t\right)}{dt}=-\left(V\left(t\right)-V_{\text{rest}}\right)+R_{m}I\left(t\right), \end{array}$$

where τ_*m*_ is the membrane time constant, *R*_*m*_ is the membrane resistance, *V*_rest_ the resting potential and *I*(*t*) is the synaptic input current. When *V*(*t*) reaches a threshold *V*_th_, the neuron generates a spike, after which *V*(*t*) resets. The liquid layer consists of a network of excitatory and inhibitory neurons with a 4:1 ratio (80% excitatory, 20% inhibitory), randomly distributed in a three-dimensional space. The connectivity probability between neurons *i* and *j* is distance-dependent, given by


(2) $$\begin{array}{l} P\left(d_{ij}\right)=C_{xx}\cdot \exp{\left(-\frac{d_{ij}}{\lambda }\right)}^{2}, \end{array}$$

where *d*_*ij*_ is the Euclidean distance between neurons, *C*_*xx*_ is the base connection probability depending on weather the pre- or post-synaptic neuron is inhibitory (I) or excitatory (E) (see Table 1), and λ is a scaling factor governing the influence of distance on connectivity. This construction creates a dynamic reservoir where input stimuli propagate through the network, generating complex temporal responses essential for processing spatiotemporal data.

**Table 1:** Parameter for the liquid configuration.

**Parameter**	**Value**
τ_*m*_	20 ms
*R*_*m*_	30
*V*_th_	15 mV
*V*_rest_	0 mV
*C*_*EE*_|*C*_*EI*_|*C*_*II*_|*C*_*IE*_	0.3|0.2|0.1|0.4
λ	2

First index in C marks the presynaptic plus second index postsynaptic neuron and (I) if its inhibitory and (E) if excitatory.

A crucial factor in achieving a rich representation of the input signal is the modeling of synaptic behavior. The used liquid configuration employs the Modified Stochastic Synaptic Model (EL-Laithy and Bogdan, 2010) that incorporates probabilistic release mechanisms and neurotransmitter dynamics, as well as responses at the postsynaptic site. This probabilistic framework, combined with the dynamics of neurotransmitter release, enables the system to explore a diverse range of states. Consequently, this enhances the fading property of the liquid and therefore the consecutive WTA computation, which can extract more spatiotemporal features from the liquid state.

### 3.2 Winner-takes-all dynamics and reward-modulated synaptic plasticity

**Figure 2:**
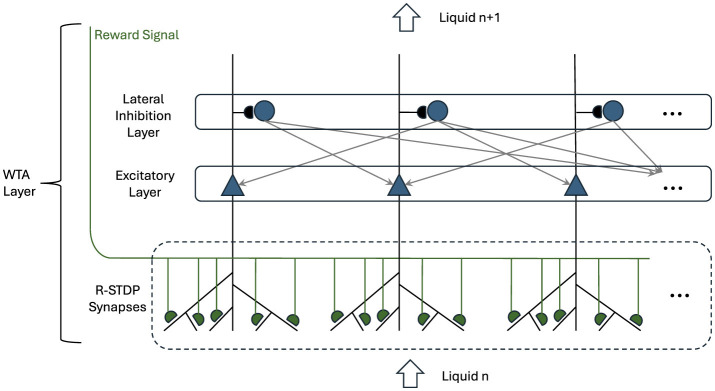
Winner-Takes-All layer with R-STDP synapses. Triangle neurons represent the winner neurons, receiving input from the preceding liquid layer. These neurons inhibit each other via round inhibitory neurons while providing their aggregated spatio temporal information to the next liquid layer. Their input synapses are governed by R-STDP and the Reward Signal is coming from the overall performance of the network.

The WTA configuration, depicted in Figure 2, incorporates both excitatory and inhibitory LIF neurons, each playing a crucial role in setting a competitive environment among the neurons. This competitive mechanism is essential for sparse activity, which is a critical aspect of efficient neural processing (Handrich et al., 2009).

In the WTA layer, the excitatory neurons are primarily tasked with receiving inputs from the preceding liquid layer through an all-to-all connection. These excitatory neurons selectively amplify the most relevant pattern based on their input. Therefore, they enhance the likelihood that the most important features of the input data are transmitted forward in the network. This selective competition ensures that only the most relevant patterns are considered, which is crucial for tasks requiring high levels of accuracy in decision-making.

Conversely, the inhibitory neurons in the WTA layer are designed to suppress the activity of less active or irrelevant excitatory neurons. This suppression is vital for ensuring that only a small, optimal subset of the most active excitatory neurons is allowed to win the competition. This winning subset then plays a significant role in shaping the outputs that contribute to downstream processing, such as classification or prediction tasks.

The synaptic connections that exist between the liquid layer and the WTA layer are governed by R-STDP synapses. In this learning mechanism, the synaptic weights between the liquid layer and the excitatory neurons in the WTA layer are dynamically updated. This process is influenced by the correlation between the spiking activities of the presynaptic neurons from the liquid layer and the postsynaptic excitatory neurons within the WTA layer. Additionally, a reward signal plays a pivotal role in this process, providing feedback that reflects the overall performance of the network on the designated task.

This reward signal acts as a guiding metric, incentivizing the network to strengthen synaptic connections that contribute to successful outcomes while diminishing those that do not. By leveraging this R-STDP learning rule, the network is able to adaptively refine its synaptic weights over time, facilitating the emergence of more robust and efficient neural pathways.

To implement the local component of the learning rule in an online fashion, each incoming presynaptic spike creates a presynaptic trace, represented as *x*_*pre*_ like in Soures and Kudithipudi (2019). This trace is mathematically defined by the following equation:


(3) $$\begin{array}{l} \frac{dx_{pre}}{dt}=\frac{x_{pre}}{\tau_{pre}}+\underset{f}{\sum }\delta \left(t-t^{f}\right), \end{array}$$

where input spikes *f* are represented by the Dirac delta function δ. This presynaptic trace effectively captures the temporal activity of the presynaptic neuron at the synapse. If no spike is arriving, the trace is decaying exponentially modulated by the constant τ_*pre*_. The continuous nature of the trace allows for a nuanced representation of the spiking history leading up to any given moment.

When the postsynaptic neuron fires, the weight of the synapse is updated based on the comparison between *x*_*pre*_ and a predefined target value *x*_*tar*_, as shown in the following equation:


(4) $$\begin{array}{l} \Delta w_{local}=\eta \left(x_{pre}-x_{tar}\right){\left(w_{max}\cdot w\right)}^{\mu }. \end{array}$$

In this equation, Δ*w*_*local*_ represents the change in the synaptic weight, η is the learning rate, *w*_*max*_ is the maximum allowable weight, and μ serves as a control parameter that determines how much the update depends on the previous weight *w*. The learning rate μ is set low, as shown in Table 2, to enable weight updates after each sample is processed. The parameter *x*_*tar*_ can be also considered as a control mechanism regulating the influence of the previous liquid to the WTA.

**Table 2:** Parameter for the learning equations.

**Parameter**	**Value**
τ_*pre*_	300 ms
η	0.0001
*w*_*max*_	1
*x*_*tar*_	20
α	15
τ_*trace*_	500 ms

STDP alone can exhibit runaway dynamics which result in synaptic strengths saturating. In order to stabilize the performance of STDP, it is necessary to use the same synaptic scaling function used in the initialization step and intrinsic plasticity. Synaptic scaling normalizes the sum of pre-synaptic connections to alpha and is applied after the weight update:


(5) $$\begin{array}{l} w_{local}=\frac{w_{i,j}}{\Sigma_{j}^{N}w_{i,j}}\cdot \alpha . \end{array}$$

To enable faster learning by crediting actions that contributed to future rewards, a eligibility trace *e*_*trace*_ is introduced. This trace acts as a bridge linking the local synaptic updates defined by Equation 5 to global delayed reward signals. The time scale of this delay is determined by τ_*trace*_, and it allows the influence of a global reward described in Section 2.3 to be distributed over time and across the relevant local synapses. This temporal distribution is vital for ensuring efficient selection of salient activities within the WTA. The equation governing the evolution of the eligibility trace is given by:


(6) $$\begin{array}{l} \frac{de_{trace}}{dt}=\frac{e_{trace}}{\tau_{trace}}+\Delta w_{local}. \end{array}$$

After each digit is processed, the eligibility trace is reflecting for each synapse between the liquid layer and the WTA the correlation between the presynaptic and postsynaptic neuron. Finally, the overall weight update for the synapses connecting the liquid layer to the WTA, incorporating feedback from the global reward signal, is expressed as follows:


(7) $$\begin{array}{l} \Delta w_{feedback}=r\cdot e_{trace}. \end{array}$$

Here, the feedback weight update Δ*w*_*feedback*_ is a product of the reward signal *r* and the value of the eligibility trace *e*_*trace*_ after the processed digit for each synapse. The reward signal *r* is calculated within the Reward-System, which compares the prediction made by the readout layer to the actual label, thereby facilitating learning based on performance feedback.

These learning rules are central to the RLSM, powering the key processes of adaptation and optimization. Through the integration of local updates, eligibility traces, and global feedback mechanisms, the system is capable of refining its synaptic weights to enhance performance on a specific task, effectively balancing exploration and exploitation in its learning strategy. All parameters for the learning process are detailed in Table 2.

### 3.3 Two approaches to feedback: forgiving and strict

Two primary approaches to feedback are extensively studied: positive reinforcement (Forgiving Feedback) and conditional synaptic decay (Strict Feedback). Positive reinforcement exclusively rewards positive outcomes, fostering a focus on achieving desired states. In contrast, conditional synaptic decay involves a gradual reduction in the strength of synaptic connections only when the reward system fails to provide a positive signal.

The positive feedback is called Forgiving Feedback and reads:


(8) $$\Delta w=\{\begin{array}{ll} k\cdot e_{trace}, & \text{if }\hat{y}=y\\ 0, & \text{otherwise}, \end{array}$$

where ŷ is the output of the logistic regression, *y* the desired spoken digit, *k* is scaling *e*_*trace*_ and *w* to the same range. That means that *r* of Equation 7 either enables learning when set to 1 or disables it when set to 0 based on the performance of the readout.

Conditional synaptic decay or Strict Feedback offers a more nuanced approach to learning compared to traditional synaptic decay, as it allows the model to maintain and strengthen synaptic connections that contribute to positive outcomes while gradually weakening connections that do not. This can improve the efficiency and accuracy of the learning process, as the model can focus on the most relevant features and patterns in the data.


(9) $$\Delta w=\{\begin{array}{ll} k\cdot e_{trace}, & \text{if }\hat{y}=y\\ \Delta w=-\gamma w, & \text{otherwise}, \end{array}$$

Equation 9 specifies that the change in synaptic weight, Δ*w*, depends on whether the predicted label matches the desired label. If they match, Δ*w* is equal to the k*e_*trace*_. If they do not match, synaptic decay occurs, and the weight decreases by an amount proportional to Δ*w* = −γ*w*, where γ is the decay factor with γ < < η.

## 4 Experiments

### 4.1 The Spiking Heidelberg Digits dataset

The SHD (Cramer et al., 2022) dataset is a benchmark specifically designed for evaluating the performance of spiking neural networks, using an audio classification task. This dataset comprises recordings of spoken digits, ranging from zero to nine, in both English and German languages. Therefore the total amount of classes is 20. The SHD dataset is structured into 8,156 training samples and 2,264 test samples. To evaluate the effectiveness of our approaches, we conduct experiments using a downsampled version of the SHD, where the original set of 700 input neurons was reduced to 70 by selecting every 10th neuron.

### 4.2 Results

In our experimental setup, the liquid structure was held constant at a fixed configuration to maintain consistency across trials. However, the initial weights of the WTA network were randomly initialized in each run, and we performed 100 independent experiments to capture the variability and assess the robustness of the approach.

The feedback mechanisms were introduced halfway through the training samples to evaluate its impact on network learning and performance. By delaying feedback activation, we aimed to examine its influence on the networks adaptability and capacity for refinement. The initial unsupervised training phase is required because the readout needs to be a rough estimator of the data. Then the feedback constantly changes the representation based on the performance of the readout. The weights are updated every sample like in stochastic gradient descent.

**Figure 3:**
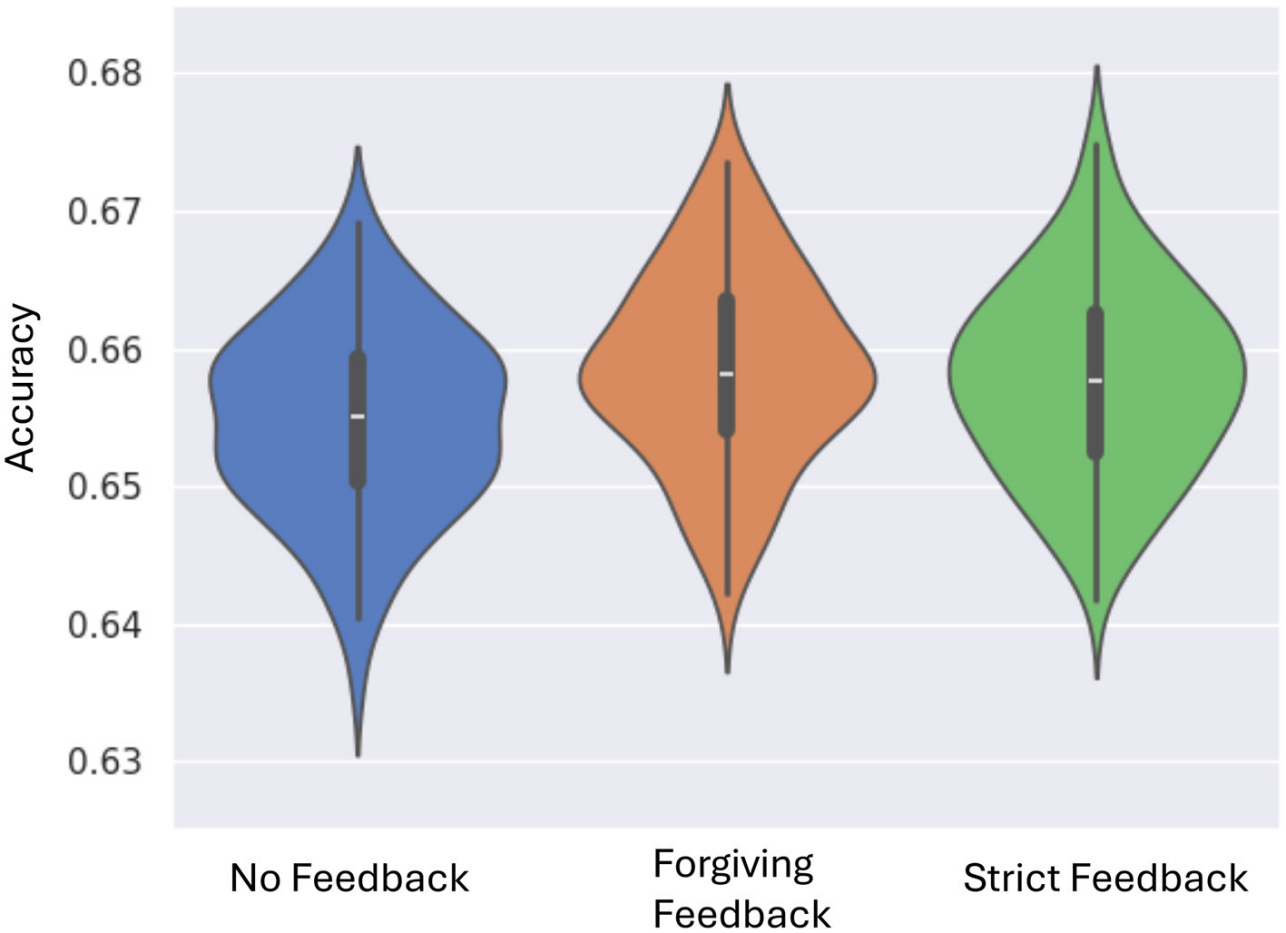
Violin plots performance across 100 experiments for each feedback mechanism. The plots show performance improvement from no feedback to forgiving and strict feedback. Most configurations converge closer to the median, indicating increased consistency with feedback.

Across 100 experimental trials, the system demonstrates a marked improvement in classification accuracy when feedback mechanisms are used compared to no feedback (Figure 3). The forgiving feedback mechanism outperforms the no-feedback baseline, achieving a maximum increase of 2.7% in performance. This result is statistically validated by the Mann-Whitney U test, with a p-value below 0.01%, confirming a significant difference in mean values and highlighting the effectiveness of forgiving feedback. The strict feedback mechanism is demonstrating comparable performance to forgiving feedback, backed by a *p-*value of 0.8%. Statistical analysis reveals no significant difference between the forgiving and strict feedback mechanisms.

**Figure 4:**
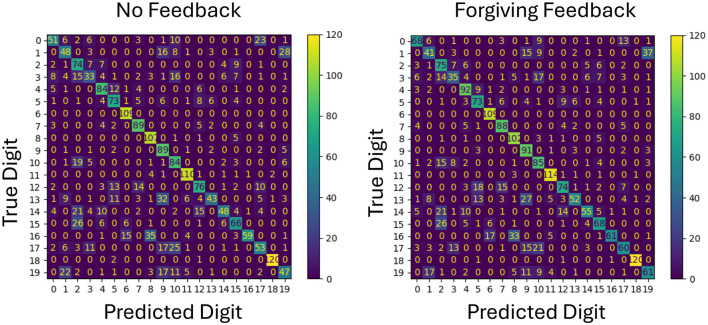
Comparison of confusion matrices for no feedback and forgiving feedback on test data. Each class represents a digit, with values on the diagonal indicating correct classifications. The diagonal values increase with forgiving feedback, showing improved accuracy for the same initial weights.

**Figure 5:**
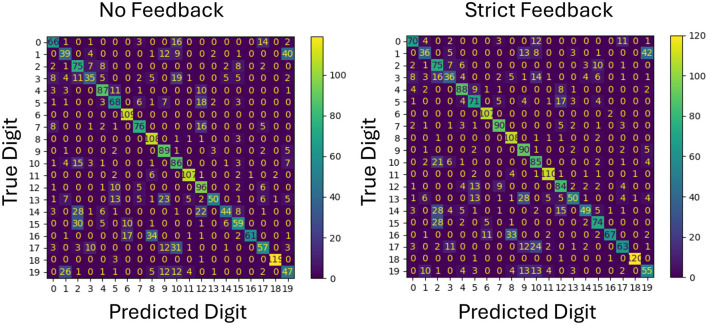
Comparison of confusion matrices for no feedback and strict feedback on test data. The diagonal values show overall improvement with strict feedback, particularly benefiting class number 7, along with most other classes.

To better understand the impact of feedback on specific classification outcomes, confusion matrices for both forgiving and strict feedback mechanisms are analyzed as shown in Figures 4, 5. This analysis provides insight into how each feedback type affects individual class predictions by using the same network configuration and the same initial weights.

The confusion matrix in Figure 4 reveals that more classes for example class 0,4 and 17 are benefiting from the additional forgiving feedback meaning that the new representations can be better separated in general.

On the other hand, the confusion matrix in Figure 5 shows a clear improvement on a single class, in this case class 7. The prediction performance of the other classes remain roughly the same.

## 5 Discussion

The comparison of the two kinds of feedback in a RLSM demonstrate that both forgiving and strict feedback mechanisms enhance classification performance compared to a no feedback baseline. Statistical analysis confirms a significant improvement for both feedback types. These results underscore the importance of feedback in SNNs, particularly when adapting to complex classification tasks. Forgiving and strict feedback mechanisms, while distinct in approach, each show effectiveness in reducing misclassification rates, suggesting that incorporating tailored feedback strategies may be valuable for optimizing model accuracy across varied classification challenges.

The forgiving feedback mechanism provides a balanced performance improvement across all classes, as evidenced by the confusion matrix analysis. This widespread reduction in misclassification rates suggests that forgiving feedback promotes a robust and generalized adaptation in the model. By allowing the system to make slight errors without harsh penalties, forgiving feedback likely facilitates smoother learning and error correction across classes. This adaptability is advantageous in environments where accuracy across all classes is essential, making forgiving feedback particularly suitable for tasks requiring consistency and generalization.

Conversely, the strict feedback mechanism demonstrates unique benefits for specific classes. Here, the inclusion of synaptic decay plays a critical role, allowing the system to effectively forget prior misclassifications, which reduces repeated errors and promotes targeted improvement for problematic classes. This focused adjustment is beneficial for classes prone to misclassification, where strict feedback helps to correct persistent error patterns. Thus, while strict feedback may not provide the broad improvement seen with forgiving feedback, its utility in enhancing performance for specific, error-prone classes is apparent. This mechanism may be particularly useful in applications where certain classifications are more critical than others and where targeted accuracy improvements are prioritized.

The absence of a statistically significant difference between forgiving and strict feedback suggests that each feedback mechanism holds comparable value in terms of overall performance enhancement. However, the unique advantages observed in the confusion matrix analysis highlight that the selection between strict and forgiving feedback should consider the specific requirements of the classification task.

## 6 Conclusion and future work

This study investigated the role of feedback mechanisms in a deep liquid state machine for spiking neural networks. We introduced strict feedback, which reinforced correct predictions while penalizing incorrect ones. Comparing this approach to a forgiving feedback strategy, we found that both significantly outperformed traditional unsupervised methods, achieving greater accuracy and adaptability. These findings underscore the importance of feedback-driven learning in optimizing spiking neural networks and contribute to the broader understanding of the role of feedback.

Future research could explore the underlying mechanisms of these feedback approaches further by analyzing the representation in the WTA. Additionally, the readout could be trained more frequently during training which might increase adaptability by forcing greater competition between the readout and the reward system. Finally, the RLSM can be evaluated across various tasks such as image recognition and video analysis to assess its generalizability and compare its performance to other spiking neural network approaches, including gradient descent-based methods.

## Data Availability

The original contributions presented in the study are included in the article/supplementary material, further inquiries can be directed to the corresponding author.
